# Incidence and risk factors of cancers in acromegaly: a Chinese single-center retrospective study

**DOI:** 10.1007/s12020-023-03447-y

**Published:** 2023-07-13

**Authors:** Tongxin Xiao, Rui Jiao, Shengmin Yang, Yi Wang, Xue Bai, Jingya Zhou, Ran Li, Linjie Wang, Hongbo Yang, Yong Yao, Kan Deng, Fengying Gong, Hui Pan, Lian Duan, Huijuan Zhu

**Affiliations:** 1 Key Laboratory of Endocrinology of National Health Commission, Department of Endocrinology, State Key Laboratory of Complex Severe and Rare Diseases, Peking Union Medical College Hospital, Chinese Academy of Medical Sciences and Peking Union Medical College, Beijing, China; 2 Eight-Year Program of Clinical Medicine, Peking Union Medical College Hospital, Peking Union Medical College, Chinese Academy of Medical Sciences, Beijing, China; 3 Department of Medical Records, Peking Union Medical College Hospital, Chinese Academy of Medical Sciences and Peking Union Medical College, Beijing, China; 4 Department of Neurosurgery, Peking Union Medical College Hospital, Chinese Academy of Medical Sciences and Peking Union Medical College, Beijing, China

**Keywords:** Acromegaly, Malignant tumors, Thyroid cancer, Risk factor, Pituitary tumors

## Abstract

**Purpose:**

To evaluate the incidence of malignancies in acromegaly and to identify risk factors for newly-diagnostic cancers, especially the excessive growth hormone (GH) and insulin-like growth factor-1 (IGF-1).

**Methods:**

A retrospective cohort including 1738 consecutive hospitalized patients with acromegaly in a single referral center between 2012 and 2020 (mean follow-up 4.3 years). A gender- and age-matched case-control study (280 patients from the cohort) was performed for risk factor analysis.

**Results:**

One hundred thirteen malignancies (67 diagnosed after acromegaly) were observed. The overall newly-diagnostic cancer risk of acromegaly was higher than the general population (standardized incidence ratio (SIR) 2.81; 95% CI 2.18–3.57). The risk of thyroid cancer (*n* = 33, SIR 21.42; 95% CI 13.74–30.08) and colorectal cancer (*n* = 8, SIR 3.17; 95% CI 1.37–6.25) was elevated. In the overall cohort, IGF-1 (ULN: 1.27 vs. 0.94, *p* = 0.057), GH (1.30 vs. 1.00 ng/ml, *p* = 0.12), and disease-controlled rate (34.9% vs. 45.9%, *p* = 0.203) at the last visit did not reach significance between patients with and without post-diagnostic cancer. In the case-control study, GH (1.80 vs. 0.90 ng/ml, *p* = 0.018) and IGF-1 (ULN: 1.27 vs. 0.91, *p* = 0.003) at the last visit were higher in patients with post-diagnostic cancers, with a lower disease-controlled rate. Elder age was a risk factor for cancer. Other metabolic comorbidities and the size of pituitary tumors were similar.

**Conclusion:**

The risk of malignancies, especially thyroid cancer, was increased in patients with acromegaly in our center. More cancer screening should be considered when managing acromegaly, especially in patients with higher posttreatment GH and IGF-1.

## Introduction

Acromegaly is usually caused by growth hormone (GH)-secreting pituitary adenoma, leading to elevated GH as well as insulin-like growth factor 1 (IGF-1) levels [1]. Chronic exposure to excessive GH and IGF-1 is likely associated with several systematic comorbidities in acromegaly, including malignant and benign tumors [2].

Malignant tumors have become the first [3, 4] or second [5, 6] most common cause of death in acromegaly. However, although the latest literature review indicated a moderately increased risk of overall cancer in acromegaly [7], whether the risk of cancer increases in acromegaly remains controversial [8, 9]. Many studies suggested a higher cancer risk in acromegaly [5, 10, 11], while some population-based or multicenter studies also indicated a similar risk to the general population [12–14]. Additionally, although preclinical research supported that elevated GH promotes tumorigenesis [15, 16], the result in clinical studies varied [4, 11, 17]. Several multicentral studies suggested that a higher posttreatment GH or IGF-1 may be related to higher cancer risk [12, 18], while some showed no difference [11, 19]. Although population-based studies may have less selection bias, a lack of acromegaly control data in detail also limited related risk factors analysis.

Debates continue on whether it is worthwhile to screen tumors earlier or more frequently in acromegaly [8]. Among all cancers, thyroid cancer and colorectal cancer are considered to be most likely to increase in many previous studies [20], as well as benign thyroid nodules [21–23] and colorectal polyps [20, 24]. However, although the latest meta-analysis indicated an elevated standardized incidence ratio (SIR) of thyroid cancer (9.2, 95% CI: 4.2–19.9), there were also large-scale studies indicating a similar risk to the general population [17, 25]. Some clinical practice guidelines have suggested colonoscopy and thyroid ultrasound for acromegaly when needed [26, 27], while some considered it nonbeneficial [8, 28]. The various cancer spectrums in different regions might contribute to conflicting results about cancer risk in different areas. By far, only one previous Chinese research evaluated the overall cancer prevalence of acromegaly, which was a cross-sectional study with 473 patients and reported a cancer prevalence of 4.3% [23].

This study aimed to investigate the cancer incidence of acromegaly in our center and to evaluate the association of risk factors related to tumorigenesis in acromegaly.

## Materials and methods

### Patients

A consecutive retrospective cohort of 1738 patients with acromegaly in Peking Union Medical College Hospital (PUMCH) between 2012/03 and 2020/12 was identified, including patients first diagnosed with acromegaly before 2012 or in other centers. Diagnosis of acromegaly was made according to clinical suspicion, hypersecretion of GH (nadir GH ≥ 1.0 ng/ml in a 75-g oral glucose tolerance test (OGTT)) and IGF-1 (>the age-adjusted upper limit of normality (ULN) of IGF-1), and pituitary lesion observed in magnetic resonance imaging (MRI) or computed tomography (CT). A histopathological diagnosis was not necessary. Thus, patients unable to tolerate or refuse to receive pituitary surgery were included. Informed consent was obtained, and this study was approved by the PUMCH ethics office (Ethical Approval Number: S-K1617).

### Medical records and telephone follow-up

We collected medical data from electronic records (from 2012/03/01 to 2022/03/14) in PUMCH. Additionally, we telephoned all 1738 patients (conducted between 2022/01 and 2022/04) to collect information including the last GH and IGF-1 measurement in any centers, treatment, cancer history, and tumor screening participation (thyroid ultrasound and colonoscopy) after the last visit to our center. In total, 1280 patients finished the telephone questionnaires, 126 received the call but failed to complete questionnaires, and 332 patients did not receive the call. For patients reporting cancer, we further collected the age at cancer diagnosis and pathological type of cancer if available. For patients with thyroid cancer, we further collected treatment and surgical details of thyroid cancer.

In the evaluation of the latest disease activity, if valid GH and IGF-1 were reported in the telephone, we applied it as the most recent results. Instead, if no detail of GH or IGF-1 was reported, we used the last GH and IGF-1 in our center. In the last measurement, patients with random or nadir GH < 1.0 ng/ml and IGF-1 ULN < 1 (Supplementary Table 1) were considered biochemical controlled acromegaly. In our center, GH and IGF-1 were determined by Siemens chemiluminescent immunoassays (Berlin, Germany). Values outside the analytic measurement range were calculated as the upper/lower limits of the measurement range (GH < 0.05 ng/ml as 0.05 ng/ml, and IGF-1 < 25 ng/ml as 25 ng/ml).

### Incidence analysis of malignancy in acromegaly

All 1738 patients were included in cancer incidence analysis. SIRs were calculated by dividing the observed malignancies after acromegaly (*n* = 67) by the expected cases in the general population (reference: the 2016 Chinese population malignancies incidence [29]). Person-years at risk: from the first visit to our center after 2012/03 to (1) the last follow-up (2022/03 for 1406 patients with telephone follow-up; the last visit to our center for 332 patients missing the call), or (2) the diagnosis of newly-diagnosed malignancy. Person-years and malignant cases were stratified according to gender and 5-year age groups. The 95% confidence intervals (CI) of SIR we computed by assuming the observed malignant cases following a Poisson distribution.

### Risk factors of malignancy in acromegaly

In the whole cohort, the last GH and IGF-1 (at least 1-year posttreatment) were analyzed. To study more risk factors, a case-control study was performed. Excluding 2 patients diagnosed or highly suspected of multiple endocrine neoplasia (MEN)-1 and 2 patients of McCune-Albright syndrome (MAS), 102 patients with cancer were included in the cancer group. For the control group, 178 patients (controls without cancers: patients with malignancy = 2:1) with >12 months posttreatment GH/IGF-1 follow-up were randomly selected from current age- (±2 years) and sex-matched cancer-free acromegaly patients.

In analyzing data at acromegaly diagnosis, patients with previous pituitary operations, pituitary radiotherapy, somatostatin analog (SSA), or dopamine agonists (DA) treatment in other centers were excluded. In the most recent status analysis, patients without 12 months posttreatment GH and IGF-1 follow-up were excluded, as well as 2 known death cases (1 with cholangiocarcinoma, 1 control).

Other risk factors at baseline were defined as (1) Diabetes mellitus (DM): diabetes history or blood glucose meeting the DM diagnosis standard in OGTT. (2) Hypertension: hypertension history or blood pressure ≥140/90 mmHg during hospitalization. (3) Hyperlipemia: hyperlipemia history, hypertriglyceridemia (TG ≥ 1.7 mmol/l), or hypercholesterolemia (TC ≥ 5.2 mmol/l, or LDL-C ≥ 3.4 mmol/l). (4) Heavy drinking: >100 grams of alcohol per week. (5) Smoking: any smoking history. (6) Secondary adrenal insufficiency: serum cortisol (8 a.m.) <3.0 μg/dl with a low or normal ACTH. (7) Secondary hypothyroidism: free thyroxine (FT4) < 0.81 ng/dl accompanied by low or normal thyroid-stimulating hormone (TSH). (8) Hypogonadotropic hypogonadism: (a) in males: serum testosterone <3.0 ng/ml, and a low or normal level (<10 IU/l) of follicle-stimulating hormone (FSH) and luteinizing hormone (LH). (b) In females: (i) in postmenopausal patients, low LH (<25 IU/l) and FSH (<40 IU/l); (ii) in premenopausal patients: menstrual disorders, a low estradiol level (<30 pg/ml) accompanied by a low or low-normal LH and FSH (<10 IU/l). Patients who had pituitary surgery or radiotherapy, thyroid surgery, or radiotherapy before the first visit were excluded from other adenohypophysis function analyses.

To evaluate cancers diagnosed in different periods, we divided them into three subgroups according to the diagnosis time of cancers and acromegaly: pre-diagnostic (cancers diagnosed 1 year before acromegaly), peri-diagnostic (cancers diagnosed 1 year before or after acromegaly), and post-diagnostic cancers (diagnosed 1 year after acromegaly). The peri-diagnostic cancers were identified because some patients were not suspected of acromegaly until they visited other departments because of malignancy. Similarly, cancers merely diagnosed after acromegaly were highly likely to exist earlier. The post-diagnostic cancers, which were more likely to develop after acromegaly, were defined to analyze the effect of posttreatment GH and IGF-1 excess.

### Statistical analysis

For continuous variables with non-normalization distribution, median [IQR (lower quartile, upper quartile)] was presented. For continuous variables with normalization distribution, mean (SD) was presented. The normality of quantitative data was examined by the Shapiro–Wilk test. For comparison between groups, continuous and categorical variables were tested by *t*-test or the Mann–Whitney *U* test and Pearson’s *χ*^2^ or Fisher’s exact test, respectively. *p* < 0.05 was considered statistically significant. Missing data were treated as missing and omitted from analysis, and no replacement was applied. All statistics were performed using R (version 4.2.2).

## Results

### Characteristics of acromegaly patients with cancers

In 1738 patients (females: *n* = 958, 55.1%), 113 primary malignancies have been diagnosed in 106 patients (5.8%; females: *n* = 80, 75.5%). Patients with malignancy had a longer follow-up period in our study (median: 6.2 vs. 4.3 years, *p* < 0.001) and were older at the last visit (mean: 54.0 ± 11.7 vs. 46.5 ± 12.7 years, *p* < 0.001). In patients with at least 1-year posttreatment GH and IGF-1 follow-up, the disease-controlled rate (45.5% vs. 45.9%) was similar between patients with cancer in any period (*n* = 88) or without cancers (*n* = 1147) (Table 1). In patients with post-diagnostic malignancies, the disease-controlled rate (34.9% vs. 45.9%, *p* = 0.20) was similar, but the most recent IGF-1 (ULN: 1.27 [0.84, 1.77] vs. 0.94 [0.75, 1.43], *p* = 0.057) tended to elevate (Table 2).

**Table 1:** Follow-up status of 1738 acromegaly patients between 2012 and 2022

Characteristics	All inpatients *N* = 1738	With malignancy^a^ *N* = 106	Without malignancy *N* = 1632	*p* value
Gender, female, *n* (%)	958 (55.1%)	80 (75.5)	878 (53.8)	<0.001
Follow-up time, years, median [IQR]^b^	4.3 [2.5, 6.5]	6.2 [3.7, 8.8]	4.3 [2.4, 6.3]	<0.001
Age at the last follow-up, years, median [IQR]	46.0 [36.8, 56.0]	53.5 [48.0, 62.8]	45.0 [36.0, 56.0]	<0.001
Controlled disease at last follow-up, *n*/*N* (%)^c^	567/1235 (45.9)	40/88 (45.5)	527/1147 (45.9)	1.00
IGF-1 ULN ≤ 1 at last follow-up, *n*/*N* (%)^c^	680/1235 (55.1)	46/88 (52.3)	634/1147 (55.3)	0.66

*IGF-1* insulin-like growth factor 1, *IGF-1 ULN* upper limit of normal of IGF-1

^a^Patient with any malignancy diagnosed either before or after the diagnosis of acromegaly

^b^Follow-up time: from acromegaly diagnosis (after 2012/03) to the latest telephone follow-up (2022/03) or the date of last medical records in our center (patients with unsuccessful telephone follow-up)

^c^In 1235 patients with more than 1-year posttreatment follow-up at the most recent GH and IGF-1 inspection

**Table 2:** Disease-controlled status of acromegaly patients with at least 1-year posttreatment follow-up

Characteristics: At the last follow-up	Without malignancy *N* = 1147	Newly-diagnostic malignancy *N* = 59	p*	Post-diagnostic malignancy *N* = 43	p**
GH, ng/ml, median [IQR]	1.00 [0.30,2.50]	1.10 [0.35,2.49]	0.50	1.30 [0.69,2.50]	0.12
IGF-1, ng/ml, median [IQR]	257 [192,384]	252 [186,431]	0.89	271 [205,434]	0.37
ULN, median [IQR]	0.94 [0.75,1.43]	0.99 [0.75,1.62]	0.42	1.27 [0.84,1.77]	0.057
Controlled disease, *n* (%)	527 (45.9)	25 (42.4)	0.69	15 (34.9)	0.20
IGF-1 ULN ≤ 1, *n* (%)	634 (55.3)	30 (50.8)	0.59	19 (44.2)	0.20

*GH* growth hormone, *IGF-1* insulin-like growth factor 1, *IGF-1 ULN* upper limit of normal of IGF-1

p*: for comparison between patients with newly-diagnostic cancer after acromegaly and patients without malignancy; p**: for comparison between patients with post-diagnostic malignancy (diagnosed at 1-year after acromegaly) and patients without malignancy

### Characteristics of patients with malignancy

**Fig. 1:**
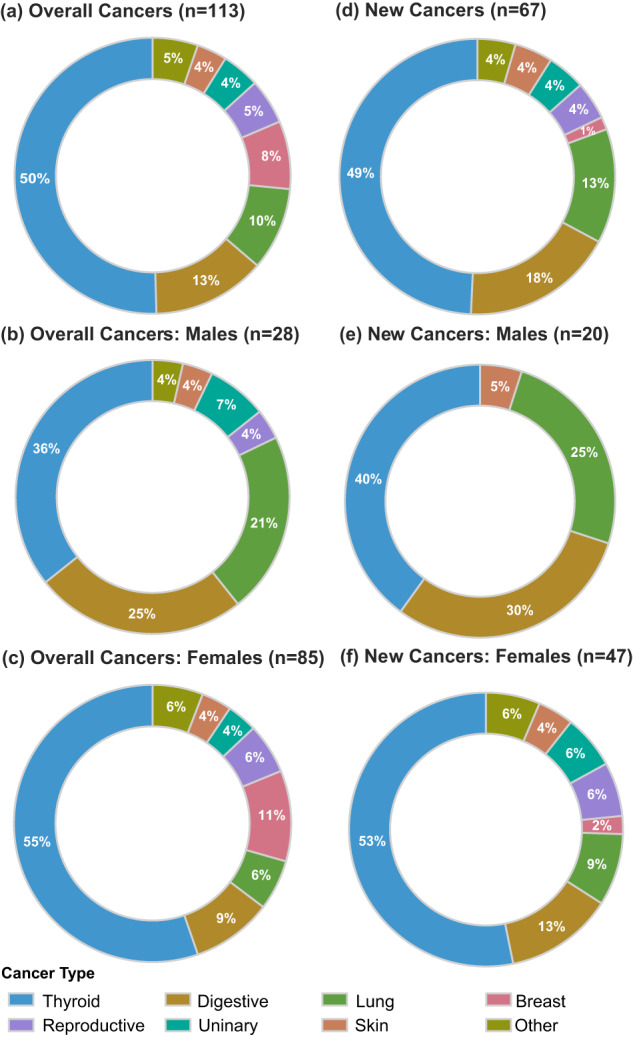
Malignancy types in acromegaly: overall cancers and newly-diagnostic cancers. **a**–**c** Overall cancers (*n* = 113, 28 males and 85 females) diagnosed before or after the diagnosis of acromegaly; **d**–**f** cancers diagnosed after the diagnosis of acromegaly (*n* = 67, 20 males and 47 females)

One hundred thirteen known malignancies (cases in females: *n* = 85, 75.2%) were recognized in all periods (Fig. 1 and Supplementary Table 2). Thyroid cancer (*n* = 57, 50.4%; 48 papillary thyroid carcinoma, 1 follicular thyroid carcinoma, 1 medullary thyroid carcinoma, and 7 unclear cases) was the most common, and malignancy of the digestive system (*n* = 15, 13.3%, including 11 colorectum’s, 1 stomach, 1 liver, 1 ampullary carcinoma, and 1 biliary system) was the second, following the lung cancers (*n* = 11), breast cancers (*n* = 9), malignancies of the reproductive system (*n* = 6, 3 endometrial, 2 cervical, 1 prostate cancer), malignancies of the urinary system (*n* = 5, 3 bladder, 1 kidney, and 1 ureter cancer), malignancies of skin (*n* = 4, 3 basal cell carcinoma, 1 squamous cell carcinoma), and lymphomas (*n* = 3). Other encountered cancers included 1 carcinoma of the parotid gland, 1 osteosarcoma, and 1 larynx carcinoma. In males, both thyroid cancer (36%) and malignancy of the digestive system (25%) were relatively common. In females, over half of the malignant cases were thyroid cancers, far surpassing the second leading digestive tract malignancies (9% overall cases, and 13% in cases diagnosed after acromegaly).

During 7277 person-years at risk (male, 3132 years; female, 4145 years) after diagnosis of acromegaly, 67 malignant cases (females: *n* = 47, 70.1%) were recognized. The overall cancer risk was significantly elevated (SIR 2.81, 95% CI: 2.18–3.57). Thyroid cancer (*n* = 33, 75.8% females) accounts for 49.3% of all cases, with a prominently elevated incidence (SIR 21.43, 95% CI: 13.74–30.08). Colorectal cancer (*n* = 8; SIR 3.17; 95% CI 1.37–6.25) was also higher in risk. The risk of lung cancers (*n* = 9; SIR 2.15; 95% CI 0.98–4.08) tended to increase. Although 9 patients had breast cancer, only 1 was diagnosed after acromegaly (Supplementary Table 2).

The mean age at cancer diagnosis was 48.5 ± 11.1 years, while it was younger (45.3 ± 9.8 years) in thyroid cancer than others (51.8 ± 11.4 years, *p* = 0.002) (Table 3). The mean diagnosis age of lung cancer (56.1 ± 10.5 years) was the eldest among cancers, with more than 3 observed cases. In addition to breast cancer (females only, mean age 49.6 ± 12.7), the cancer diagnosis age was similar in males and females.

**Table 3:** Comparison between patients with thyroid cancers and other malignant tumors

Characteristics	Overall *N* = 113	Thyroid *N* = 57	Non-thyroid *N* = 56	*p* value (Thyroid vs. Others)	Colorectal *N* = 11	Lung *N* = 11	Breast *N* = 9	Others *N* = 25
Gender, female, *n* (%)	85 (75.2)	47 (82.5)	38 (67.9)	0.11	6 (54.5)	5 (45.5)	9 (100)	18 (72)
Time to malignancy, years, median [IQR]	0.4 [−1.6,4.8]	0.3 [−1.0,4.1]	0.8 [−2.7,5.6]	0.71	4.4 [−0.3,10.2]	4.0 [0.9,5.6]	−4.0 [−5.7, −2.0]*	0.4 [−2.5,5.5]
Age at malignancy, years, mean (SD)	48.5 (11.1)	45.3 (9.8)	51.8 (11.4)	0.002	51.2 (9.7)	56.1(10.5)*	49.5 (12.6)	51.0 (12.3)
Age at acromegaly, year, mean (SD)	46.7 (11.7)	44.1 (10.8)	49.4 (12.0)	0.019	45.3 (13.2)	51.3 (11.4)	54.1 (8.9)	49.0 (12.5)
Controlled acromegaly: >1-year follow-up, *n*/*N* (%)	40/88 (45.5)	23/48 (47.9)	17/40 (42.5)	0.77	4/8 (50.0)	2/8 (25.0)	3/5 (60.0)	8/19 (42.1)
Controlled acromegaly in post-diagnostic cancers, *n*/*N*(%)	15/47 (31.9)	5/19 (26.3)	10/28 (35.7)	0.72	3/7 (42.9)	2/8 (25.0)	1/1 (100)	4/12 (33.3)

Post-diagnostic cancers: malignant tumors observed at least 1-year after the diagnosis of acromegaly. Patients with two primary malignant tumors were counted twice in two different sites of cancer

*: *p* < 0.05 in subgroup analysis when compared to thyroid cancers

Seven patients (2 male, 5 female) were diagnosed with two primary cancers (Supplementary Table 3). Among them, 2 cases (1 thyroid cancer, 1 lymphoma) were diagnosed during the peri-diagnostic period of acromegaly, while 4 cases were post-diagnostic (2 lung cancers, 1 thyroid cancer, and 1 colon cancer), and 8 cases were pre-diagnostic (3 thyroid cancers, 2 breast cancers, 2 lung cancers, 1 bladder cancer). Compared to patients with one malignant tumor, patients with two primary malignancies were similar in age at diagnosis of acromegaly or malignancies.

**Fig. 2:**
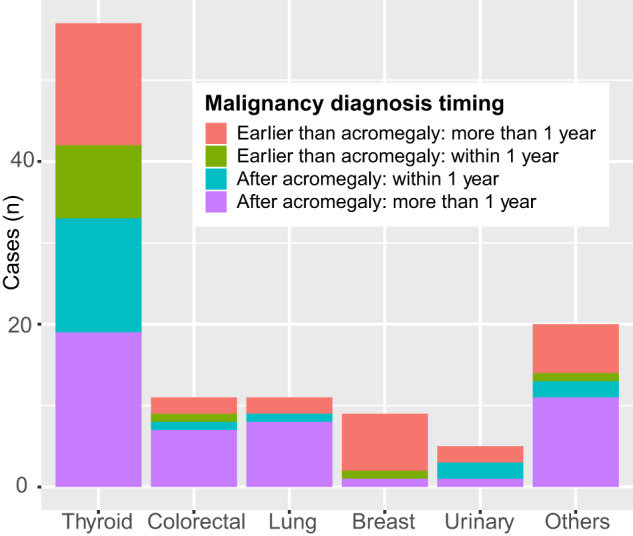
Diagnosed time of different malignant tumors: earlier or later than acromegaly

Thirty-two (28.3%) cancers were recognized in the peri-diagnostic period of acromegaly, among which 23 (71.9%) were thyroid cancers (Fig. 2). In 46 malignancies diagnosed before acromegaly, only 10 (8.8%) cancers (in 7 patients) were likely diagnosed earlier than the onset time of acromegaly (5 thyroids, 3 breast, 1 cervical cancer, and 1 lymphoma) (Supplementary Table 4). These patients were at a younger age of cancer (42.7 ± 7.6 to 49.1 ± 11.2 years, *p* = 0.031) and elder age at the diagnosis of acromegaly (53.5 ± 6.0 to 46.2 ± 11.9 years, *p* = 0.018).

### Risk factors of malignancies in acromegaly: the case-control study

Patients with (*n* = 102) or without cancers (*n* = 178) were sex- and age-matched here (about 75% females and 52 years at baseline). Patients with post-diagnostic cancers were younger at the diagnosis of acromegaly (median: 42.5–48.5 years, *p* = 0.027). The acromegaly onset also tended to be younger in patients with post-diagnostic cancers (36.6 ± 11.5 to 40.1 ± 11.1 years, *p* = 0.090) (Table 4).

**Table 4:** Baseline characteristics of acromegaly with and without cancer: sex- and age-matched case-control

Characteristics	Without cancer *N* = 178	With cancer *N* = 102	*p* value*	Post-diagnostic cancer *N* = 44	*p* value**
Gender, female, *n* (%)	133 (74.7)	77 (75.5)	1.00	32 (72.7)	0.94
Age at acromegaly onset, years, mean (SD)	40.1 (11.1)	39.9 (11.0)	0.92	36.6 (11.5)	0.090
Age at acromegaly diagnosis, years, median [IQR]	48.5 [41.0,55.8],	48.0 [39.0,54.0]	0.32	42.5 [34.8,53.0]	0.027
Delay diagnosis, years, median [IQR]	6.0 [3.0,10.0]	5.0 [3.0,8.5]	0.15	5.0 [3.0,8.0]	0.42
Age at last visit, median [IQR]	52.6 [46.6,60.8]	51.9 [46.1,61.1]	0.98	51.5 [45.3,61.0]	0.97
Treatment					
Surgery, *n* (%)	172 (96.6)	92 (90.2)	0.049	42 (95.5)	0.66
Re-operation, *n* (%)	9 (5.06)	11 (11.0)	0.11	10 (23.3)	0.001
Radiotherapy, *n* (%)	23 (12.9)	13 (13.0)	1.00	10 (23.3)	0.14
SSA, *n* (%)	39 (21.9)	36 (36.0)	0.016	19 (44.2)	0.005
DA, *n* (%)	17 (9.55)	10 (10.0)	1.00	5 (11.6)	0.78
Cancer familiarity, *n* (%)	15 (8.43)	17 (16.7)	0.059	10 (22.7)	0.014
Smoking, *n* (%)	22 (12.4)	14 (13.7)	0.89	4 (9.09)	0.73
Drunk, *n* (%)	14 (7.87)	8 (7.84)	1.00	4 (9.09)	0.76
BMI, kg/m^2^, mean (SD)	27.4 (4.6)^a^	26.0 (3.71)^b^	0.14	25.9 (4.63)^c^	0.25
Comorbidity					
Diabetes mellitus, *n* (%)	70 (39.3)	40 (39.2)	1.00	17 (38.6)	1.00
Hypertension, *n* (%)	86 (48.3)	45 (45.5)^d^	0.74	18 (41.9)^e^	0.56
Hyperlipidemia, *n* (%)	74 (41.6)	44 (47.8)^f^	0.39	18 (48.6)^g^	0.54
Radiologic evaluation of pituitary adenoma					
Knosp grade, *n* (%)	*n* = 169	*n* = 82	0.72	*n* = 32	0.38
Knosp 0	75 (44.4)	32 (39.0)		10 (31.2)	
Knosp 1–2	53 (31.4)	28 (34.1)		13 (40.6)	
Knosp 3–4	41 (24.3)	22 (26.8)		9 (28.1)	
Tumor size	*n* = 174	*n* = 83	0.80	*n* = 33	0.72
Microadenoma (<10 mm), *n*(%)	28 (16.1)	11 (13.1)		4 (12.1)	
Macroadenoma (10–40 mm), *n*(%)	142 (81.6)	61 (85.7)		28 (84.9)	
Giant adenoma (>40 mm), *n*(%)	4 (2.3)	1 (1.2)		1 (3.0)	
The largest dimension, median [IQR]	15.6 [12.0,21.0]	17.0 [11.2,21.5]	0.82	17.0 [12.0,23.0]	0.48
Adenoma immunopathology: Ki-67, %, median [IQR]	2.0 [1.0,3.0]^h^	1.0[1.0,3.0]^i^	0.57	1.5 [1.0,3.0]^j^	0.56

Age at last visit: at the last follow-up in telephone or the last visit to our center

*SSA* somatostatin analog, *DA* dopamine agonists, *BMI* body mass index

p*: Comparison between patients with cancer and patients without cancer; p**: Comparison between patients with newly-onset cancer and patients without cancer

^a^39

^b^38

^c^18

^d^99

^e^43

^f^92

^g^32

^h^147

^i^60

^j^22

Regards treatment, more patients with cancer received SSA (44.2% in post-diagnostic cancers, 36.0% in overall cancers, vs. 21.9% in controls, *p* < 0.05). Patients with cancers tended to receive more re-operation (11.0% vs. 5.1%, *p* = 0.110), especially in patients with post-diagnostic cancers (23.3%, *p* = 0.001). Radiotherapy and DA treatment were similar (Table 4).

More family history of cancer was noticed in patients with post-diagnostic cancers (22.7%, *p* = 0.014), while no difference was observed in the specific distribution of the type of familial malignancies (Supplementary Table 5). Smoking or drinking habits, metabolism-related comorbidities (Table 4), and other adenohypophysis functions (Supplementary Table 6) were similar at baseline. The pituitary adenomas also presented a similar aggressiveness (Knosp 3–4: 26.8% vs. 24.3%; Ki-67: 2% vs. 1%, *p* = 0.57) and size (microadenoma: 13.1% vs. 16.1%; largest dimension: 18.1 ± 9.0 vs. 17.6 ± 8.4 mm).

At the diagnosis of acromegaly, random GH, nadir GH, and IGF-1 levels were similar in patients with or without cancers (Table 5). In the last visit, for overall patients with cancers, GH (median: 1.50 vs. 0.90 ng/ml, *p* = 0.017) and IGF-1 (IGF-1, median: 252 vs. 214 ng/ml, *p* = 0.050; ULN, 1.01 vs. 0.91, *p* = 0.070) tended to elevate, but the acromegaly-controlled rate was similar (46.4% vs. 52.8%). Compared with patients without cancer, those with post-diagnostic cancer (*n* = 41) had a higher GH (median: 1.80 vs. 0.90 ng/ml, *p* = 0.018), and IGF-1 level (median: 285 vs. 214 ng/ml, *p* = 0.003; ULN, 1.27 vs. 0.91, *p* = 0.003), as well as a lower acromegaly-controlled rate (34.1% vs. 52.8%, *p* = 0.048).

**Table 5:** GH and IGF-1 in acromegaly patients with or without malignancy: at diagnosis and the last follow-up

Characteristics	Without cancer	Cancer: overall	*p* value*	Cancer: newly-diagnostic	*p* value**	Cancer: post-diagnostic	*p* value***
At diagnosis of acromegaly, median [IQR]	*N* = 171	*N* = 81	–	*N* = 50	–	*N* = 32	–
GH, ng/ml	11.30 [6.45,21.95]	14.20 [5.97,28.40]	0.47	17.80 [5.78,30.60]	0.21	14.70 [5.40,29.52]	0.56
GH nadir, ng/ml	8.67 [5.11,16.40]^a^	10.80 [4.96,19.65]^b^	0.38	10.60 [4.77,21.75]^c^	0.39	9.42 [4.32,19.40]^d^	0.97
IGF-1, ng/ml	782 [647,949]	733 [579,928]	0.23	789 [620,1008]	0.93	795 [592,994]	0.86
ULN	3.14 [2.59,3.87]	3.00 [2.58,3.54]	0.30	3.06 [2.68,3.54]	0.69	3.12 [2.56,3.54]	0.53
At the last follow-up, median [IQR]	*N* = 178	*N* = 102	–	*N* = 64	–	*N* = 44	–
GH, ng/ml	0.90 [0.40,2.50]	1.50 [0.80,2.70]	0.008	1.80 [0.85,2.95]	0.014	1.80 [0.98,2.52]	0.012
IGF-1, ng/ml	214 [159,330]	253 [179,432]	0.011	275 [194,450]	0.005	287 [206,442]	0.001
ULN	0.91 [0.68,1.25]	1.11 [0.77,1.73]	0.015	1.20 [0.77,1.93]	0.010	1.28 [0.87,1.91]	0.001
Controlled disease, *n* (%)	94 (52.8)	42 (41.2)	0.080	24 (37.5)	0.051	14 (31.8)	0.020
ULN ≤ 1, *n* (%)	106 (59.6)	46 (45.1)	0.027	28 (43.8)	0.042	17 (38.6)	0.027
At the last follow-up > 1-year, median [IQR]	*N* = 178	*N* = 84	–	*N* = 57	–	*N* = 41	–
GH, ng/ml	0.90 [0.40,2.50]	1.50 [0.80,2.80]	0.017	1.70 [0.80,3.20]	0.031	1.80 [0.90,2.60]	0.018
IGF-1, ng/ml	214 [159,330]	252 [178,390]	0.050	253 [179,433]	0.040	285 [206,434]	0.003
ULN	0.91 [0.68,1.25]	1.01 [0.72,1.65]	0.070	1.04 [0.73,1.70]	0.069	1.27 [0.85,1.83]	0.003
Controlled disease, *n* (%)	94 (52.8)	39 (46.4)	0.41	24 (42.1)	0.21	14 (34.1)	0.048
ULN ≤ 1, *n* (%)	106 (59.6)	42 (50.0)	0.19	28 (49.1)	0.22	17 (41.5)	0.054

*GH* growth hormone, *IGF-1* insulin-like growth factor 1, *IGF-1 ULN* upper limit of normal of IGF-1

p*: Comparison between patients with cancer and patients without cancer; p**: Comparison between patients with newly-diagnostic cancer (diagnosed after acromegaly) and patients without cancer; p***: Comparison between patients with post-diagnostic cancer (diagnosed 1 year after acromegaly) and patients without cancer

^a^147

^b^68

^c^39

^d^26

### Risk factors of thyroid cancers

Nineteen thyroid cancers were diagnosed in the post-diagnostic period of acromegaly (15 females, 79.0%). In the cohort, similarly to the overall cancers, the most recent GH, IGF-1, and acromegaly-controlled rates were similar (Supplementary Table 7). In the case-control section, 18 patients (excluded 1 with MEN-1) with post-diagnostic thyroid cancers were younger at the onset (median: 29.5 vs. 41.0 years, *p* = 0.006) and diagnosis of acromegaly (median: 38.0 vs. 48.5 years, *p* < 0.001). IGF-1 was elevated in the last visit (ULN, median: 1.51 vs. 0.91, *p* = 0.034) (Supplementary Table 8). Thyroid function at the acromegaly baseline was similar (Supplementary Table 9). Seventeen out of 40 papillary thyroid carcinoma cases with known diameter were microcarcinoma (diameter <1 cm), and 10 out of 51 thyroid cancer cases received Iodine-131 treatment after thyroidectomy (Supplementary Table 10).

## Discussion

Our study showed a higher risk of overall cancers, especially thyroid and colorectal cancer, consistent with many previous studies [30]. Considering the increasing age of patients and the trend of increasing cancer risk in the general population [29, 31], the period prevalence of cancer in our cohort is also likely to increase in further follow-up.

Although some research still indicated a similar risk of thyroid cancer in acromegaly [17, 32, 33], the difference in cancer spectrum in various regions might partially explain these conflicts. In our center, thyroid cancers accounted for 49.7% of new cancers, and females were at higher risk, which was similar to large-scale research in Korea [19], as well as several small-scale studies in China [23, 34] and Japan [35]. This might be a regional commonality in East Asia. Another interesting topic is the potential overdiagnosis of thyroid cancer. Although the incidence of thyroid cancer increased in recent years worldwide (partially because of modern diagnostic techniques), the mortality rate remained low [36]. This increase in thyroid cancer incidence is most prominent in females aged 35–64 worldwide [36], which was also similar in our study (82% females; mean age: 45.3). According to a previous epidemiologic study, the overdiagnosis rate of thyroid cancer might be even over 80% in some urban area, which might not cause symptoms or death without surgery [37]. Similarly, no death has been observed in patients with thyroid cancer in our cohort yet. In our cohort, about 10% of patients with thyroid cancer had to receive neck dissection and about 20% of patients received I-131 after surgery. Although the more severe cases did not represent the majority and the mortality was not affected, the cases receiving more extensive treatment could not be disregarded easily either. Since acromegaly is an endocrinology-related disease and has long been suspected to increase tumor risks, patients with acromegaly are more likely to receive thyroid ultrasound, leading to selection bias. Considering thyroid ultrasound inspection has also become more common in health checks in China’s general population, the fairly high SIR of thyroid cancer did indicate a higher risk. However, the potential burden and harm might not be as significant as the SIR showed. In short, even for thyroid cancer, which is considered to be the most acknowledged type of cancer with an elevated risk in acromegaly, there is still controversy regarding whether the risk greatly increases or if it is elevated worldwide.

The risk of colorectal cancer was moderately elevated in our cohort, consistent with many studies [38]. Most were diagnosed after acromegaly, even when the follow-up time was relatively short. The age at diagnosis of colorectal cancer was 51 on average (from 33 to 66 years) in our center, with 4 of 11 diagnosed younger than 50 (the traditionally strongly recommended starting age for inspection in population of average risk [39]). By far, the recommended starting age for regular colonoscopy screening in acromegaly has not been unified in different consensus. Although we considered an earlier colorectal tumor screening in acromegaly might benefit, it should be noted that whether long-term active acromegaly increases the risk of colorectal cancer remained controversial. Similar uncertainty exists regarding the risk of breast cancer and prostate cancer in acromegaly.

Most previous studies indicated no association between cancer and GH or IGF-1 at the diagnosis of acromegaly [11, 40], which was also supported by our study. In the case-control section, patients with cancers diagnosed after acromegaly had a higher GH, IGF-1 and the disease-uncontrolled rate in the last visit. Additionally, more patients with post-diagnostic cancers received re-operation and SSA treatment, which also indicated more refractory acromegaly in patients with cancers.

Notably, in the analysis of the overall acromegaly cohort in our center, differences between the most recent GH, IGF-1, and remission rates were less significant than in the case-control section. First, it could be partially explained by the difference in the age of controls in these two sections. In the case-control section, patients diagnosed with acromegaly at a younger age tended to have a higher risk of developing cancers, which might indicate a longer disease duration. A possibly varying degree of effect of excess GH and IGF-1 might also promote varying in younger and older patients might also affect. Second, selection bias could contribute to the difference. Since the control group in the case-control study was with at least 1-year follow-up, their characteristics might be different from patients lost to follow-up. This inconsistency in the most recent GH and IGF-1 in two sections of our study might also partially present the conflicting results on the association between cancers and GH or IGF-1 in many previous studies [18, 19]. Any differences in study design, data source, follow-up period, and frequency might potentially affect the significance of the analysis of GH or IGF-1.

The overall disease-controlled rate of acromegaly in our cohort was 45.9% when setting GH < 1.0 ng/ml and IGF-1 ULN ≤ 1.0 as the cutoff, which was relatively low among recent large-scale studies (46–75%) [4, 11, 23]. It is likely to be underestimated by applying these criteria in our center since many patients with approximately normal IGF-1 refused to receive OGTT, which left only random GH (without nadir GH) for the analysis during follow-up. Based on IGF-1, the overall remission rate would be 55% in patients. Combining that random fasting GH is probably a poor predictor of IGF-1 in acromegaly [41], IGF-1 (especially ULN) was more stable and might be more likely to reflect the risk of cancers in acromegaly.

Other common cancer risk factors mostly showed no difference in our study. A family history of cancer was likely a risk factor for cancer, as in the general population. Our cohort had relatively few smoking or drinking habits, leading to insufficient statistical power. The size and aggressiveness of pituitary GH-secreting adenoma, other adenohypophysis functions, and metabolic comorbidities were also similar.

There are several limitations to our study. First, the mean follow-up period (4.3 years) was still short for observing post-diagnostic cancers, while the loss of follow-up rate of the cohort was around 20%. Although we performed a latest telephone follow-up, it did not reach 19.1% of patients. Considering many patients were followed up in other local centers after initial treatment, the loss of follow-up rate in a retrospective cohort was acceptable. We plan to reduce this dropout rate through a more robust electronic follow-up system in the future study of this consecutive cohort of acromegaly. Second, we analyzed detailed clinical and biochemical data in a case-control study but not in the overall cohort. Because of the nature of the retrospective study, some present illness history and previous treatments were not described in detail, mostly in patients first diagnosed or followed up in other centers. Although patients in the control group were randomly sampled from age- and sex-matched ones, the inevitable selection bias and confounding could affect the results. Lastly, no national medical registry system of acromegaly could provide systemic diagnosis coding of post-diagnostic comorbidities, including cancers. We had to conduct telephone follow-ups to study the current status, which was inevitably less reliable than accessing the original medical records. Since patients with cancers were generally at a higher risk of mortality, the prevalence of malignant tumors might be higher in patients lost to follow-up because of death. This could lead to recall bias and an underestimation of cancer risk after the diagnosis of acromegaly. A long-term prospective study with more frequent and systemic follow-up on this consecutive cohort will be continued, enabling us to learn more about the timing of loss to follow-up (possibly indicating the death).

In conclusion, in our center, overall cancer incidence increased in acromegaly, especially thyroid and colorectal cancers. Thyroid cancer incidence increasing without change in mortality may be partially due to active surveillance, but some still need timely treatment. A successful acromegaly control might decrease the excessive risk of cancer in the posttreatment period of acromegaly and benefit the prognosis.

### Supplementary Information

supplementary_tables_revised

STROBE-checklist_revised

## Data Availability

The clinical data and codes that support the findings of the current study are not publicly available due to ethics requirements but are available from the corresponding author upon reasonable request.
