# Supplementary material for: Incidence and risk factors of cancers in acromegaly: a Chinese single-center retrospective study
Source: Endocrine. 2023 Jul 13;82(2):368–78. doi: 10.1007/s12020-023-03447-y (PMC10543525; doi:10.1007/s12020-023-03447-y)
Supplement: Supplementary file 1 — supplementary_tables_revised [file 12020_2023_3447_MOESM1_ESM.docx]

**Information**

Title: Incidence and risk factors of cancers in acromegaly: a Chinese single-center retrospective study

Author names: Tongxin Xiao, Rui Jiao, Shengmin Yang, Yi Wang, Xue Bai, Jingya Zhou, Ran Li, Linjie Wang, Hongbo Yang, Yong Yao, Kan Deng, Fengying Gong, Hui Pan, Lian Duan, Huijuan Zhu

**Corresponding Author:**

Lian Duan *, Huijuan Zhu

Department of Endocrinology, Peking Union Medical College Hospital, Peking Union Medical College, Chinese Academy of Medical Sciences

E-mail: [duanlianpumc@163.com](mailto:duanlianpumc@163.com), shengxin2004@163.com

**Supplementary Table 1:** Reference of the age-adjusted upper limit of normality of IGF-1

| **Age (years)** | **ULN of IGF-1 (ng/ml)** |
| --- | --- |
| 18-24 | 358 |
| 25-29 | 329 |
| 30-34 | 307 |
| 35-39 | 284 |
| 40-44 | 267 |
| 45-49 | 252 |
| 50-54 | 238 |
| 55-59 | 225 |
| 60-64 | 212 |
| 65-69 | 200 |
| 70 and elder | 177 |

Reference range of IGF-1 in our center between 2012/03 and 2022/01. IGF-1: insulin-like growth factor 1; ULN: upper limit of normality.

**Supplementary Table 2:** Malignancy types and incidence in acromegaly: overall cancers and newly-diagnostic cancers

| **Sites of Malignancy** | **Overall（n=1738）** | | | **Male（n=780）** | | | **Female（n=958）** | | |
| --- | --- | --- | --- | --- | --- | --- | --- | --- | --- |
|  | **Total** | **Newly-diagnostic** | **SIR**  **(95% CI)** | **Total** | **Newly-diagnostic** | **SIR**  **(95% CI)** | **Total** | **Newly-diagnostic** | **SIR (95%CI)** |
| **Overall** | 113 | 67 | 2.81  (2.18-3.57） | 28 | 20 | 2.18  (1.31-3.40) | 85 | 47 | 3.70  (2.72-4.92) |
| **Thyroid** | 57 | 33 ^a^ | 21.42  (13.74-30.08) | 10 | 8 ^a^ | 23.78  (9.56-49.00) | 47 | 25 | 19.06  (12.33-28.13） |
| **Colon-rectum** | 11 | 8 | 3.17  (1.37-6.25) | 5 | 4 | 2.86  (0.78-7.32) | 6 | 4 | 3.36  (0.92-8.60) |
| **Lung** | 11 | 9 | 2.15  (0.98-4.08) | 6 | 5 | 3.62  (1.18-8.45) | 5 | 4 | 2.16  (0.59-5.53) |
| **Breast** | 9 | 1 | … | … | … | … | 9 | 1 | … |
| **Skin** | 4 | 3 | … | 1 | 1 | … | 3 | 2 | … |
| **Bladder** | 3 | 1 | … | 2 | 0 | … | 1 | 1 | … |
| **Lymphoma** | 3 | 1 | … | … | … | … | 3 | 1 | … |
| **Cervix** | 3 | 1 | … | … | … | … | 3 | 1 | … |
| **Uterus** | 2 | 2 | … | … | … | … | 2 | 2 | … |
| **Kidney and Ureter** | 2 | 2 | … | … | … | … | 2 | 2 | … |
| **Prostate** | 1 | 0 | … | 1 | 0 | … | … | … | … |
| **Stomach** | 1 | 1 | … | 1 | 1 | … | … | … | … |
| **Liver** | 1 | 1 | … | 1 | 1 | … | … | … | … |
| **Gallbladder** | 1 | 1 | … | … | … | … | 1 | 1 | … |
| **Ampulla of Vater** | 1 | 1 | … | … | … | … | 1 | 1 | … |
| **Larynx** | 1 | 0 | … | 1 | 0 | … | … | … | … |
| **Bone** | 1 | 1 | … | … | … | … | 1 | 1 | … |
| **Parotid** | 1 | 1 | … | … | … | … | 1 | 1 | … |

Only newly-diagnosed cancers were included in SIR calculation. Newly-diagnostic cancers: malignant tumors observed after the diagnosis of acromegaly. In the subgroups with less than 3 observed cases, SIR analysis was not performed. ^a^ A patient diagnosed with thyroid cancer in 2001 and acromegaly in 1980 was included in the number of newly diagnosed cases, but not in the SIR calculation. Abbreviations: SIR, standard incidence ratio; CI, confidence interval.

**Supplementary table 3:** Patients with two primary malignancies

| **Case** | **Age at acromegaly diagnosis** | **The first malignancy** | | | **The second malignancy** | | |
| --- | --- | --- | --- | --- | --- | --- | --- |
|  |  | **Site** | **Time to Acromegaly (year)** | **Age (year)** | **Site** | **Time to acromegaly (year)** | **Age**  **(year)** |
| 1(F) | 51.0 | Thyroid | -6.3 | 44.7 | Lung | -2.3 | 48.7 |
| 2(F) | 57.4 | Thyroid | -1.7 | 55.7 | Lung | 4.0 | 61.4 |
| 3(F) | 40.3 | Thyroid | -5.4 | 34.9 | Colon | 6.4 | 46.7 |
| 4(F) | 67.8 | Breast | -4.4 | 63.5 | Thyroid | -0.4 | 67.5 |
| 5(M) | 28.4 | Thyroid | 21.0 | 49.4 | Lung | 39.2 | 67.6 |
| 6(M) | 46.1 | Lung | -12.5 | 33.5 | Bladder | -2.5 | 43.6 |
| 7(F) | 35.6 | Breast | -6.6 | 29.0 | Lymphoma | -0.8 | 34.8 |

Time to acromegaly: a negative time indicated that the malignant tumor was diagnosed before acromegaly. Case 6 was a patient diagnosed with McCune-Albright syndrome. F, female; M, male.

**Supplementary table 4.** 113 cases of malignant tumors in 106 patients with acromegaly

| No. | Gender | Age: Acromegaly (years) | Age: cancer (years) | Site of cancer | Time to cancer (years) | Controlled acromegaly at the last visit | Cancer before the onset of acromegaly |
| --- | --- | --- | --- | --- | --- | --- | --- |
| 1 | F | 22.6 | 30.2 | Others | 7.6 | NO | NO |
| 2 | M | 22.7 | 45.5 | Colorectal | 22.8 | YES | NO |
| 3 | M | 24.7 | 24.5 | Thyroid | -0.1 | YES | NO |
| 4 | F | 25.2 | 25.9 | Thyroid | 0.7 | NO | NO |
| 5 | F | 26.6 | 37.6 | Thyroid | 10.9 | NO | NO |
| 6 | F | 27.0 | 27.1 | Thyroid | 0.1 | YES | NO |
| 7 | F | 27.1 | 29.8 | Others | 2.7 | YES | NO |
| 8 | M | 28.1 | 28.4 | Thyroid | 0.3 | NO | NO |
| 9a | M | 28.4 | 67.6 | Lung | 39.2 | NO | NO |
| 9b | M | 28.4 | 49.4 | Thyroid | 21.0 | NO | NO |
| 10 | M | 29.4 | 33.3 | Colorectal | 3.9 | YES | NO |
| 11 | F | 29.7 | 39.7 | Thyroid | 10.0 | NO | NO |
| 12 | F | 29.8 | 29.1 | Thyroid | -0.7 | NO | NO |
| 13 | F | 31.2 | 37.7 | Thyroid | 6.6 | YES | NO |
| 14 | M | 31.4 | 36.2 | Thyroid | 4.8 | NO | NO |
| 15 | F | 32.5 | 33.2 | Thyroid | 0.6 | NO | NO |
| 16 | F | 32.5 | 38.0 | Others | 5.5 | NO | NO |
| 17 | F | 32.7 | 49.1 | Others | 16.3 | NO | NO |
| 18 | F | 33.5 | 37.7 | Thyroid | 4.2 | NO | NO |
| 19 | F | 33.6 | 33.2 | Thyroid | -0.3 | NO | NO |
| 20a | F | 35.6 | 34.8 | Others | -0.8 | YES | NO |
| 20b | F | 35.6 | 29.0 | Breast | -6.6 | YES | YES |
| 21 | F | 35.8 | 37.0 | Thyroid | 1.3 | NO | NO |
| 22 | F | 35.9 | 45.3 | Thyroid | 9.4 | NO | NO |
| 23 | F | 36.9 | 35.8 | Thyroid | -1.1 | YES | NO |
| 24 | F | 37.3 | 42.6 | Thyroid | 5.4 | NO | NO |
| 25 | F | 38.6 | 37.8 | Colorectal | -0.8 | NO | NO |
| 26 | M | 38.7 | 39.6 | Thyroid | 0.8 | YES | NO |
| 27 | M | 39.2 | 43.3 | Thyroid | 4.1 | YES | NO |
| 28 | F | 39.5 | 53.0 | Colorectal | 13.5 | YES | NO |
| 29 | F | 39.5 | 39.9 | Thyroid | 0.4 | YES | NO |
| 30 | F | 40.3 | 40.8 | Thyroid | 0.5 | YES | NO |
| 31a | F | 40.3 | 46.7 | Colorectal | 6.4 | NO | NO |
| 31b | F | 40.3 | 34.9 | Thyroid | -5.4 | NO | YES |
| 32 | F | 40.5 | 39.6 | Thyroid | -0.9 | YES | NO |
| 33 | F | 41.1 | 45.5 | Thyroid | 4.4 | NO | NO |
| 34 | F | 41.2 | 55.3 | Colorectal | 14.1 | NO | NO |
| 35 | F | 41.4 | 43.1 | Thyroid | 1.7 | NO | NO |
| 36 | F | 41.4 | 52.8 | Thyroid | 11.4 | YES | NO |
| 37 | M | 41.6 | 41.6 | Thyroid | 0.1 | YES | NO |
| 38 | M | 41.7 | 46.0 | Others | 4.3 | YES | NO |
| 39 | F | 42.5 | 49.0 | Thyroid | 6.6 | YES | NO |
| 40 | F | 42.6 | 46.7 | Others | 4.1 | NO | NO |
| 41 | F | 43.0 | 48.4 | Lung | 5.4 | YES | NO |
| 42 | F | 43.8 | 43.8 | Thyroid | 0.0 | NO | NO |
| 43 | F | 44.2 | 44.6 | Thyroid | 0.4 | NO | NO |
| 44 | M | 44.7 | 44.9 | Others | 0.3 | YES | NO |
| 45 | F | 45.0 | 33.8 | Breast | -11.2 | YES | YES |
| 46 | F | 45.2 | 43.6 | Thyroid | -1.6 | YES | NO |
| 47a | M | 46.1 | 43.6 | Urinary | -2.5 | NO | NO |
| 47b | M | 46.1 | 33.5 | Lung | -12.5 | NO | NO |
| 48 | M | 46.2 | 45.9 | Thyroid | -0.4 | YES | NO |
| 49 | F | 46.5 | 42.7 | Thyroid | -3.8 | NO | NO |
| 50 | F | 46.7 | 43.8 | Thyroid | -2.9 | NO | NO |
| 51 | M | 46.7 | 44.3 | Others | -2.3 | NO | NO |
| 52 | F | 46.9 | 48.4 | Breast | 1.5 | YES | NO |
| 53 | F | 46.9 | 47.0 | Urinary | 0.1 | NO | NO |
| 54 | M | 47.7 | 57.1 | Lung | 9.4 | NO | NO |
| 55 | M | 47.7 | 53.4 | Lung | 5.7 | NO | NO |
| 56 | F | 48.1 | 48.5 | Others | 0.4 | NO | NO |
| 57 | F | 48.7 | 48.2 | Thyroid | -0.5 | YES | NO |
| 58 | M | 48.7 | 53.1 | Colorectal | 4.4 | NO | NO |
| 59 | F | 49.9 | 58.8 | Thyroid | 8.9 | NO | NO |
| 60 | F | 50.1 | 46.5 | Thyroid | -3.7 | NO | YES |
| 61 | M | 50.1 | 47.1 | Urinary | -3.0 | NO | NO |
| 62 | F | 50.6 | 49.4 | Thyroid | -1.2 | NO | NO |
| 63 | F | 50.6 | 47.2 | Breast | -3.4 | YES | NO |
| 64 | F | 50.7 | 49.7 | Thyroid | -1.0 | NO | NO |
| 65a | F | 51.0 | 48.7 | Lung | -2.3 | YES | NO |
| 65b | F | 51.0 | 44.7 | Thyroid | -6.3 | YES | YES |
| 66 | F | 51.4 | 44.8 | Others | -6.6 | NO | YES |
| 67 | M | 51.6 | 53.1 | Lung | 1.5 | NO | NO |
| 68 | F | 51.6 | 57.0 | Lung | 5.5 | NO | NO |
| 69 | F | 51.9 | 52.3 | Thyroid | 0.4 | NO | NO |
| 70 | F | 52.0 | 51.0 | Breast | -1.0 | NO | NO |
| 71 | F | 52.3 | 52.7 | Thyroid | 0.4 | YES | NO |
| 72 | F | 52.7 | 52.8 | Urinary | 0.1 | YES | NO |
| 73 | F | 52.8 | 48.9 | Breast | -4.0 | NO | YES |
| 74 | F | 52.8 | 42.7 | Thyroid | -10.1 | NO | YES |
| 75 | M | 53.0 | 58.0 | Thyroid | 5.0 | NO | NO |
| 76 | F | 53.0 | 55.5 | Thyroid | 2.5 | NO | NO |
| 77 | F | 53.1 | 65.1 | Others | 12.0 | NO | NO |
| 78 | F | 53.2 | 61.3 | Others | 8.1 | YES | NO |
| 79 | M | 54.0 | 54.1 | Colorectal | 0.1 | NO | NO |
| 80 | F | 54.5 | 58.6 | Thyroid | 4.1 | YES | NO |
| 81 | F | 55.0 | 50.5 | Thyroid | -4.6 | YES | NO |
| 82 | F | 55.2 | 50.1 | Others | -5.1 | YES | NO |
| 83 | F | 55.2 | 60.2 | Others | 5.0 | NO | NO |
| 84 | F | 55.5 | 57.7 | Thyroid | 2.2 | NO | NO |
| 85 | F | 55.7 | 55.9 | Thyroid | 0.2 | YES | NO |
| 86 | F | 57.0 | 55.5 | Thyroid | -1.5 | NO | NO |
| 87 | F | 57.3 | 56.3 | Thyroid | -1.0 | NO | NO |
| 88a | F | 57.4 | 61.4 | Lung | 4.0 | NO | NO |
| 88b | F | 57.4 | 55.7 | Thyroid | -1.7 | NO | NO |
| 89 | M | 58.2 | 65.0 | Others | 6.8 | YES | NO |
| 90 | F | 59.1 | 50.1 | Thyroid | -9.0 | NO | YES |
| 91 | F | 59.5 | 66.3 | Colorectal | 6.8 | NO | NO |
| 92 | M | 60.6 | 61.5 | Thyroid | 1.0 | NO | NO |
| 93 | F | 60.7 | 59.6 | Thyroid | -1.1 | NO | NO |
| 94 | F | 60.9 | 55.3 | Breast | -5.7 | NO | NO |
| 95 | F | 61.3 | 56.5 | Others | -4.9 | YES | NO |
| 96 | F | 61.3 | 60.6 | Thyroid | -0.7 | YES | NO |
| 97 | M | 62.1 | 58.1 | Colorectal | -4.0 | NO | NO |
| 98 | F | 62.2 | 59.7 | Colorectal | -2.5 | NO | NO |
| 99 | F | 63.3 | 51.5 | Others | -11.8 | NO | YES |
| 100 | F | 63.3 | 78.5 | Others | 15.2 | NO | NO |
| 101 | F | 65.5 | 67.6 | Lung | 2.1 | NO | NO |
| 102a | F | 67.8 | 67.5 | Thyroid | -0.4 | YES | NO |
| 102b | F | 67.8 | 63.5 | Breast | -4.4 | YES | NO |
| 103 | F | 68.5 | 69.7 | Urinary | 1.2 | NO | NO |
| 104 | M | 69.2 | 69.4 | Lung | 0.2 | NO | NO |
| 105 | F | 70.6 | 68.6 | Breast | -2.0 | NO | NO |
| 106 | M | 73.1 | 68.8 | Others | -4.3 | YES | NO |

The same number with a and b indicates two primary cancers in one patient. F, female; M, male.

**Supplementary table 5:** Detailed family history of cancers in patients with acromegaly

|  | **Overall n=280** | **Without cancer**  **n=178** | **With cancer**  **n=102** | **p value** |
| --- | --- | --- | --- | --- |
| Familial malignancy history | 32 (11.4) | 15 (8.4) | 17 (16.7) | 0.059 |
| Type of familial malignancy, n (%) ^a^ | 41 | 19 | 22 | 0.311 |
| Lung cancer | 10 (24.4) | 7 (36.8) | 3 (13.6) |  |
| Colorectal cancer | 6 (14.6) | 2 (10.5) | 4 (18.2) |  |
| Liver cancer | 6 (14.6) | 2 (10.5) | 4 (18.2) |  |
| Breast | 4 (9.8) | 0 (0.0) | 4 (18.2) |  |
| Stomach cancer | 3 (7.3) | 1 (5.3) | 2 (9.1) |  |
| Glioma | 2 (4.9) | 1 (5.3) | 1 (4.5) |  |
| Bladder | 1 (2.4) | 1 (5.3) | 0 (0.0) |  |
| Cholangiocarcinoma | 1 (2.4) | 1 (5.3) | 0 (0.0) |  |
| Gallbladder cancer | 1 (2.4) | 1 (5.3) | 0 (0.0) |  |
| Leukemia | 1 (2.4) | 1 (5.3) | 0 (0.0) |  |
| Nasopharyngeal cancer | 1 (2.4) | 1 (5.3) | 0 (0.0) |  |
| Osteosarcoma | 1 (2.4) | 0 (0.0) | 1 (4.5) |  |
| Pancreatic cancer | 1 (2.4) | 1 (5.3) | 0 (0.0) |  |
| Renal cancer | 1 (2.4) | 0 (0.0) | 1 (4.5) |  |
| Thyroid cancer | 1 (2.4) | 0 (0.0) | 1 (4.5) |  |
| Undefined ^b^ | 1 (2.4) | 0 (0.0) | 1 (4.5) |  |

^a^ 9 patients (5 with cancer and 4 without cancer) had a family history of malignancies in multiple first- or second-degree relatives. Thus, 41 specific familial malignancies were analyzed. ^b^ 1 patient with colon cancer had a familial history of his father passing away due to malignancy, but the specific type remained unclear.

**Supplementary Table 6:** Comparison of adenohypophysis functions at baseline between patients with post-diagnostic cancers and patients without cancer

| **Hormones at baseline**  **median [IQR]** | **Reference** | | **Without cancer**  **n=169** | **Post-diagnostic cancers n=52** | **p-value** |
| --- | --- | --- | --- | --- | --- |
| **PRL, ng/ml** | | F: <30  M: 2.6-13.1 | 11.9 [6.98,24.4] ^a^ | 12.0 [8.11,28.6] ^b^ | 0.63 |
| **FSH, IU/L** | | * | 12.3 [5.34;40.2] ^c^ | 6.36 [3.92;21.0] ^d^ | 0.03 |
| **LH, IU/L** | | * | 6.03 [2.83;15.6] ^c^ | 3.95 [1.91;9.67] ^d^ | 0.056 |
| **E2 (female), pg/ml** | | * | 21.5 [15.0;48.6] ^e^ | 30.0 [13.5;72.5] ^f^ | 0.51 |
| **T (male), ng/ml** | | M: 3.50-7.81 | 1.89 [1.26;2.86] ^g^ | 2.73 [2.08;3.20] ^h^ | 0.074 |
| **FT3, pg/ml** | | 1.8-4.1 | 3.03 [2.74,3.37] ^i^ | 3.08 [2.80;3.50] ^j^ | 0.51 |
| **FT4, ng/dl** | | 0.81-1.89 | 1.15 [1.06;1.27] ^i^ | 1.18 [1.02;1.35] ^j^ | 0.56 |
| **T3, ng/ml** | | 0.66-1.92 | 1.14 [1.00;1.29] ^k^ | 1.07 [0.91;1.29] ^l^ | 0.49 |
| **T4, μg/dl** | | 4.3-12.5 | 8.50 [7.27;9.79] ^k^ | 8.13 [6.86;9.62] ^l^ | 0.45 |
| **TSH, μIU/ml** | | 0.38-4.34 | 1.40 [0.78;2.11] ^i^ | 1.26 [0.82;1.71] ^j^ | 0.50 |
| **F,** **μg/dl** | | 4.0-22.3 | 12.8 [9.7,16.1] ^m^ | 12.5 [8.0,15.1] ^n^ | 0.33 |
| **ACTH, pg/ml** | | 0-46 | 29.3 [18.4,45.5] ^o^ | 28.6 [21.6,36.9] ^p^ | 0.74 |
| **Secondary adrenal insufficiency** | | - | 3/166 (1.8%) | 1/52 (1.9%) | 1.00 |
| **Secondary hypothyroidism** | | - | 5/167 (3.0%) | 2/51 (3.9%) | 0.67 |
| **Hypogonadotropic hypogonadism** | | - | 43/130 (33.1%) | 16/42 (38.1%) | 0.68 |

Patients who received thyroid operation before the diagnosis of acromegaly were excluded from the thyroid functions. E2, estradiol; FSH, follicle stimulating hormone; FT3, free triiodothyronine; FT4, free thyroxine; LH, luteinizing hormone; PRL, prolactin; T, testosterone; T3, Triiodothyronine; T4, thyroxine; TSH, thyroid-stimulating hormone.

^a^ n=155; ^b^ n=50; ^c^ n=134; ^d^ n=44; ^e^ n=94; ^f^ n=30; ^g^ n=43; ^h^ n=17; ^i^ n=166; ^j^ n=51; ^k^ n=143; ^l^ n=43; ^m^ n=166; ^n^ n=52; ^o^ n=130; ^p^ n=45. *The reference of FSH: in males, 1.27-19.26IU/L; in females: menopause >40IU/L, follicular phase <10.0IU/L, ovulatory phase 4.54-30.34IU/L, luteal phase 1.65-0.66IU/L; The reference of LH: in males, 1.24-8.62IU/L; in females: menopause 10.87-58.64IU/L, follicular phase 2.12-10.89IU/L, ovulatory phase 19.18-103.03IU/L, luteal phase 1.20-12.86IU/L; The reference of E2 in females: menopause <25pg/ml, follicular phase 22-115pg/ml, ovulatory phase 32-517pg/ml, luteal phase 37-246pg/ml.

**Supplementary Table 7:** Comparison of characteristics between patients with thyroid cancers and patients without malignancy: in patients followed up at least 1 year

| **Characteristics:**  **At the last follow-up** | **Without Malignancy**  **n=1147** | | **Thyroid cancer: post-diagnostic**  **n=19** | **p-value** | |
| --- | --- | --- | --- | --- | --- |
| **Gender, females, n (%)** | 638 (55.6) | | 15 (79.0) | 0.072 | |
| **Age, years, median [IQR]** | 45.0 [36.0,55.0] | | 52.0 [43.0,58.5] | 0.031 | |
| **GH, ng/ml, median [IQR]** | 1.00 [0.30,2.50] | 1.30 [0.69,2.55] | | | 0.26 |
| **IGF-1, ng/ml, median [IQR]** | 257 [192,384] | 364 [206,470] | | | 0.17 |
| **ULN, median [IQR]** | 0.94 [0.75,1.43] | 1.50 [0.84,1.93] | | | 0.074 |
| **Controlled acromegaly, n (%)** | 527 (45.9) | 5 (26.3) | | | 0.14 |
| **Controlled acromegaly: IGF-1 ULN<=1, n (%)** | 634 (55.3) | 7 (36.9) | | | 0.17 |

IGF-1, insulin-like growth factor 1; ULN, the upper limit of normality of IGF-1. Post-diagnostic cancers: cancers diagnosed at least 1 year after the diagnosis of acromegaly.

**Supplementary table 8:** Comparison of characteristics and acromegaly-controlled status between patients with post-diagnostic thyroid cancers and patients without cancer

| **Characteristics** | **Without Cancer**  **n=178** | **Post-diagnostic thyroid cancers**  **n=18** | **p-value** |
| --- | --- | --- | --- |
| **gender, female, n (%)** | 133 (74.7) | 14 (77.8) | 1.00 |
| **Age at acromegaly onset, years, median [IQR]** | 41.0 [31.2,48.0] | 29.5 [27.0,35.8] | 0.006 |
| **Age at acromegaly diagnosis, years, median [IQR]** | 48.5 [41.0,55.8] | 38.0 [31.2,41.0] | <0.001 |
| **Delay diagnosis, years, median [IQR]** | 6.0 [3.0,10.0] | 4.5 [3.0,8.0] | 0.42 |
| **Cancer familiarity, n (%)** | 15 (8.4) | 4 (22.2) | 0.08 |
| **Comorbidity** | | | |
| **Diabetes Mellitus, n (%)** | 70 (39.3) | 3 (22.2) | 0.24 |
| **Hypertension, n (%)** | 86 (48.3) | 8 (47.1) | 1.00 |
| **Hyperlipidemia, n (%)** | 74 (41.6) | 5 (33.3) | 0.73 |
| **Radiologic evaluation** | | | |
| **Knosp grade, n (%)** | n=169 | n=11 | 0.034 |
| **Knosp 0** | 75 (44.4) | 1 (9.1) |  |
| **Knosp 1-2** | 53 (31.4) | 7 (63.6) |  |
| **Knosp 3-4** | 41 (24.3) | 3 (27.3) |  |
| **Tumor size** | n=174 | n=12 | 0.24 |
| **microadenoma (<10mm), n(%)** | 28 (16.1) | 1 (8.3) |  |
| **macroadenoma (10-30mm), n(%)** | 142 (81.6) | 10 (83.4) |  |
| **giant adenoma (>40mm), n(%)** | 4 (2.3) | 1 (8.3) |  |
| **Largest dimension, median [IQR]** | 15.6 [12.0,21.0] | 20.4 [16.1,30.0] | 0.14 |
| **At the diagnosis of acromegaly, median [IQR]** | n=171 | n=14 |  |
| **GH, ng/ml** | 11.30 [6.45,21.95] | 22.30 [5.25,53.50] | 0.36 |
| **IGF-1, ng/ml** | 782 [647,949] | 988 [793,1075] | 0.084 |
| **IGF-1/ULN** | 3.14 [2.59,3.87] | 3.48 [2.82,3.60] | 0.47 |
| **At the last follow-up, median [IQR]** | n=178 | n=18 |  |
| **GH, ng/ml** | 0.90 [0.40,2.50] | 1.25 [0.69,2.58] | 0.35 |
| **IGF-1, ng/ml** | 214 [159,330] | 367 [205,480] | 0.016 |
| **IGF-1/ULN** | 0.91 [0.68,1.25] | 1.51 [0.83,1.93] | 0.034 |
| **Controlled disease, n (%)** | 94 (52.8) | 5 (27.8) | 0.076 |

GH, growth hormone; IGF-1, insulin-like growth factor 1; ULN, the upper limit of normality of IGF-1. 1 patient with MEN-1 and post-diagnostic thyroid cancer was excluded.

**Supplementary table 9:** Comparison of thyroid function between patients with thyroid cancer after acromegaly and patients without cancer

| Hormones | Without malignancy | Thyroid cancer: post-diagnostic | p-value |
| --- | --- | --- | --- |
| At the diagnosis of acromegaly | N=167 | N=32 | - |
| FT3, pg/ml, median [IQR] | 3.03 [2.74,3.36] | 2.98 [2.81,3.14] | 0.36 |
| FT4, ng/dl, median [IQR] | 1.14 [1.06,1.26] | 1.11 [0.98,1.27] | 0.47 |
| T3, ng/ml, median [IQR] | 1.14 [1.00,1.29] ^a^ | 1.07 [0.90,1.22] ^b^ | 0.24 |
| T4, μg/dl, median [IQR] | 8.49 [7.18,9.72] ^a^ | 7.55 [6.49,9.02] ^b^ | 0.095 |
| TSH, μIU/ml, median [IQR] | 1.39 [0.77,2.06] | 1.21 [0.69,1.68] ^c^ | 0.27 |
| At the last visit or before the diagnosis of thyroid cancer | N=168 | N=26 | - |
| FT3, pg/ml, median [IQR] | 2.95 [2.69,3.24] | 2.99 [2.67,3.24] | 0.71 |
| FT4, ng/dl, mean (SD) | 1.20 (0.21) | 1.12 (0.19) | 0.035 |
| T3, ng/ml, median [IQR] | 1.05 [0.93,1.20] ^d^ | 1.04 [0.92,1.23] ^e^ | 0.88 |
| T4, μg/dl, mean (SD) | 8.37 (1.89) ^d^ | 8.00 (1.76) ^e^ | 0.39 |
| TSH, μIU/ml, median [IQR] | 1.60 [0.95,2.56] | 1.43 [0.79,2.04] ^f^ | 0.25 |

FT3, free triiodothyronine; FT4, free thyroxine; T, testosterone; T3, Triiodothyronine; T4, thyroxine; TSH, thyroid-stimulating hormone. Patients who received thyroid operation before the diagnosis of acromegaly were excluded. ^a^ n=144; ^b^ n=25; ^c^ n=30; ^d^ n=148; ^e^ n=20.

**Supplementary table 10:** Pathology and follow-up treatment of thyroid cancers in patients with acromegaly

| **Characteristics** | **Thyroid cancer (n=57)** |
| --- | --- |
| Pathology, n (%) ^a^ |  |
| PTC | 48 (96.0) |
| Follicular | 1 (2.0) |
| Medullary + PTC | 1 (2.0) |
| Preoperative lymph node metastasis in ultrasound, n (%) ^b^ | 9 (19.1) |
| Lymph node metastasis in pathology, n (%) ^b^ |  |
| None | 16 (34.0) |
| Central compartment metastasis only | 15 (31.9) |
| Lateral compartment metastasis | 5 (10.6) |
| Supraclavicular nodes metastasis | 1 (2.1) |
| Others ^f^ | 1 (2.1) |
| Unclear | 9 (19.1) |
| Maximal diameter of PTC, n (%) ^c^ |  |
| <1cm | 17 (39.5) |
| 1-2cm | 20 (46.5) |
| 2-4cm | 3 (7.0) |
| Unclear | 1 (2.3) |
| The extent of thyroidectomy, n (%) ^d^ |  |
| Bilateral total thyroidectomy | 26 (51.0) |
| partial thyroidectomy | 20 (39.2) |
| total thyroidectomy with neck dissection | 5 (9.8) |
| Treatment after thyroidectomy, n (%) ^e^ |  |
| No treatment other than surgery | 38 (79.2) |
| Iodine-131 | 9 (18.8) |
| Reoperation | 0 (0) |
| Iodine-131 and Reoperation | 1 (2.1) |
| Persistent voice disturbance after thyroidectomy, n (%) | 3 (6.0) |

^a^ n=50, ^b^ n=47, ^c^ n=43, ^d^ n=51, ^e^ n=48, ^f^ cancer infiltration in the perinodal fibrous tissue of central compartment lymph nodes. PTC: Papillary thyroid carcinoma.
